# Sex offender registration and notification act with adolescents adjudicated for illegal sexual behavior: a therapeutic jurisprudence perspective

**DOI:** 10.3389/fpsyt.2023.1160922

**Published:** 2023-04-25

**Authors:** Apryl Alexander, John Michael Falligant, Cory Marchi, Erica Floding, Marissa Jennings

**Affiliations:** ^1^University of North Carolina at Charlotte, Charlotte, NC, United States; ^2^Johns Hopkins Medicine, Johns Hopkins University, Baltimore, MD, United States; ^3^Kennedy Krieger Institute, Baltimore, MD, United States; ^4^University of Denver, Denver, CO, United States

**Keywords:** juvenile deliquency, policy, registry, sexual offending, therapeutic jurisprudence

## Abstract

Adolescents adjudicated for illegal sexual behavior (AISB) are subjected to the same Sex Offender Registration and Notification Act (SORNA) policies as adults with sexual offense histories despite current research documenting their relatively low likelihood of recidivism. Therapeutic jurisprudence is a framework which suggests the law should value psychological well-being and strive to avoid imposing anti-therapeutic consequences. The purpose of this article is to analyze the use of SORNA policies with AISB from a therapeutic jurisprudence perspective. Given the current literature documenting the collateral consequences of SORNA on AISB and their families and the lack of efficacy in reducing recidivism, we argue SORNA should not be applied to children and adolescents. We conclude with a discussion of future directions for the juvenile justice system and public policy reform.

## Introduction

The consequences of engaging in illegal sexual behavior as a juvenile can be lifelong. However, the law treats juveniles who engage in illegal sexual behavior as adults who have sexually offended–meaning that juveniles (like adults) are subject to sex offense registry and notification laws that can impact the rest of their lives. Adolescents adjudicated for illegal sexual behavior (AISB) are responsible for 36% of sex offenses committed against children each year (1) and 15% of forcible rapes (2) in the United States. AISB typically begin “hands-on” offending between 10 and 14 years of age (1, 3). Furthermore, AISB have more child victims than adults who sexually offend, as the proportion of child victims under the age of 12 is 59% for AISB compared to 39% for adults who sexually offend (1). There is great heterogeneity in the type of offenses committed by AISB, ranging from relatively low risk behaviors (e.g., indecent exposure, sexting) to high risk (e.g., rape). Juveniles tend to be amenable to treatment and exhibit relatively low recidivism rates when compared with adults. Given that evidence-based interventions result in lower rates of sexual recidivism (4–7), many states require AISB to receive psychological treatment for their illegal sexual behavior (7). A meta-analysis of 33,783 cases of AISB revealed the weighted mean base rate for sexual recidivism was ~5% at a mean follow-up period of 59 months (8). In fact, 33% of those studies, which were conducted in the last 15 years, reported a mean sexual recidivism of 2.75%, highlighting a decreasing trend in recidivism. AISB exhibit higher recidivism rates for *nonsexual offenses* when compared to sexual offenses (9). Indeed, the 10-year sexual recidivism rate for AISB who received treatment is identical to sexual recidivism rates for youth previously adjudicated for *non-sexual* offenses (5). Another study found a 6.2% recidivism rate upon a follow-up period of 15.76 years for those who were adjudicated as youth (10).

Adolescents are often subjected to the same Sex Offender Registration Notification Act (SORNA) requirements as adults, which includes placing AISB on public sex offense registries and enforcing community notification and residency restriction policies. Proponents of registration of juveniles argue AISB pose a unique threat to the public through re-offending (11). However, recent research has questioned these assumptions about individuals who sexually offend. For instance, pervasive myths include: (1) individuals who commit sexual offenses and their motivations are all the same (i.e., they are a homogenous population); (2) almost all will re-offend; and (3) treatment is ineffective (12). These assumptions are so prevalent that mental health professionals, law enforcement personnel, and the public continue to believe them (13–17). Several scholars have cited “moral panic,” often due to extensive media attention dedicated to coverage of isolated incidents involving those who engage in repeat offending who have committed a homicide, as the cause legislation changes (12, 18–21). Moreover, the SORNA requirements are associated with iatrogenic community, educational, and vocational consequences, including mandatory quarterly or annual registration and updated registration when they change residences (22, 23).

Outside of the United States, several countries are being pressured into implementing public sex offending registries similar to the United States’ registry (24). At present, several countries mandate sex offense registration including Argentina, Australia, Bermuda, Canada, France, Germany, Ireland, Jamaica, South Africa, South Korea, Taiwan, and the United Kingdom. However, not all countries impose a public registry or community notification system (e.g., Argentina, Australia, France, Germany). Although countries like Australia, Canada, and New Zealand have registries for law enforcement agencies to track and monitor individuals who have sexually offended, each has considered extending the registry to the public. The aim of this paper is to analyze and critique the downward application of SORNA laws and policies to AISB in the United States using behavioral science research and a therapeutic jurisprudence framework.

## Therapeutic jurisprudence

Therapeutic jurisprudence (TJ) has successfully been used as a therapeutic framework for addressing important policy issues within the criminal and juvenile justice systems (25, 26). David Wexler first introduced the notion of TJ by defining it as “the study of the role of the law as a therapeutic agent” [(27), p. 43]. The aim of TJ is to analyze both the therapeutic and anti-therapeutic aspects and consequences of legal rules, procedures, and decision-making from legal actors (i.e., lawyers, judges; (27–30)). This framework emphasizes the importance of examining the well-being of individuals involved in the legal system. Therapeutic jurisprudence suggests the laws should value psychological well-being, bring about healing and wellness, and strive to avoid imposing anti-therapeutic consequences when possible (28, 31, 32). Although TJ does not propose well-being should be the law’s ultimate role (33–35), it does assert laws should not cause harm (33). Instituting the principles of TJ into the juvenile justice system is ideal, particularly when attempting to mitigate the anti-therapeutic effects on vulnerable adolescents (31). This perspective aligns with the *parens patriae* doctrine of the juvenile court system, which allows the courts to act on behalf of the well-being of children and vulnerable citizens. Further, therapeutic jurisprudence encourages the use of behavioral science research to improve upon the understanding of the law, the anti-therapeutic effects of laws, and examine ways to maximize the law’s therapeutic potential (32).

## Sex Offender Registration and Notification Act (SORNA)

The Sex Offender Registration and Notification Act (SORNA) established the public registry in the United States. On July 27, 2006, the 25th anniversary of the abduction and murder of a 6-year-old Adam Walsh in Hollywood, Florida, the *Adam Walsh Child Protection and Safety Act of 2006* (better known as the Adam Walsh Act) was signed into law by President George W. Bush. The intention of the law was to protect children from sexual exploitation and violent crime. The Adam Walsh Act (AWA) was also proposed to prevent child abuse and child pornography, promote Internet safety, and honor the memory of Adam Walsh and other child crime victims. The AWA established minimum standards for registration and community notifications in the United States and its territories. The minimum standards included the development of a public online registry for jurisdictions, as well as reporting the types of information required to be included in the registration (i.e., duration of registration requirements, periodic in-person verifications, and the duty to notify the public about registration requirements for individuals with sexual offense histories). Title I of the AWA established SORNA which created the sex offender registration and notification (SORN) laws, which established registration requirements for state, territorial, and tribal jurisdictions. SORN laws allow law enforcement agencies to track, supervise, and monitor those who have committed a sexual offense. Citizens also have access to information about these individuals through community notification laws.

State policies also vary regarding the extent with which they comply with SORNA standards (36). Currently, 18 states, four territories, and 119 tribes in the United States have substantially implemented SORN requirements (37). SORN laws classify persons with sexual offense histories into three risk tiers based on the nature of the offense. These classifications all have specific lengths of registration someone adjudicated or convicted of a sexual offense within that tier will be subject to and individuals are required to register based solely on the offense committed without regard to a determination of future risk. Tier I classification requirements are the least stringent, only defined as an offense that does not meet Tier II or III criteria, and those meeting Tier I classification are excluded from registration. Tier II classification is defined as offenses that are subjected to more than 1 year of imprisonment and involves a minor victim. Tier II offenses have a registration requirement of at least 25 years. Tier III offenses, which are more violent in nature (i.e., aggravated sexual abuse of someone under age 18, kidnapping of a minor) and involve a minor that is <13 years old, require lifetime registration. Most AISB included under SORNA would qualify to be placed on the Tier III classification.

Researchers have applied the TJ framework to SORNA and adults with sexual offense histories (38–43). SORN laws may not be particularly effective at decreasing sexual recidivism among adults with sexual offense histories (see (44)). Few statutes and laws pertaining to adults with sexual offense histories are aligned with one’s potential for transformation, change, and rehabilitation; therefore, the SORN laws are anti-therapeutic (38, 41). In fact, Levenson et al. (45) called for evidence-based registry reform, which better aligns with the principles of a TJ framework. A TJ approach would explore whether the application of SORN laws with AISB have beneficial or therapeutic effects on AISB while protecting public safety.

### SORN and AISB

Though TJ has been discussed as an important framework for adults who committed sexual offenses, rarely has the framework been discussed for AISB. The introduction of the SORNA also included adolescents at least 14 years of age and stipulated the inclusion of juveniles on the national registration and notification database. SORN laws often require AISB whom have been adjudicated for certain serious sexual offenses (e.g., aggravated sexual abuse, possession of child sexual exploitation material, sex trafficking) to be treated in the same manner as adults with sexual offense histories. AISB must also comply with SORN requirements, such as registering with law enforcement and exposing their personal identifying information to the public (46). Youth who offended after their 14th birthday and who were adjudicated delinquent for a crime comparable to or more severe than aggravated sexual abuse as defined by federal law (Sexual Abuse Act of 1986) against a child under 12 would be included in the registry. Thus, adolescents 14 years or older adjudicated for sexual offenses could be subjected to long-term public registration (25 years to life).

These policies were extended to juveniles with the belief that AISB were also at significant risk of re-offending, highly resistant to rehabilitation, and have more in common with adults with sexual offense histories than general delinquent youth (47, 48). Critics have argued against the implementation and application of SORN laws with adolescents given the goal of SORN is to prevent repeat offending by individuals who have already been convicted of sexual crimes (23). As previously noted, the sexual recidivism rate for AISB is relatively low. AISB tend to be a heterogeneous population with varying personal characteristics and treatment needs (11, 49); thus, SORN inaccurately categorizes many adolescents as high risk and the lifelong registration requirements are often excessive. Further, in some jurisdictions, SORN requires registration for relatively low-risk behaviors that may not be considered sexual offenses (e.g., indecent exposure, public urination).

Much of the extant research on SORN as applied to juveniles has evaluated whether or not SORNA policies have reduced sexual or nonsexual recidivism. To date, no research has indicated that SORN reduces sexual recidivism reductions nor increases public safety (11, 15, 50–56). Community notification laws were developed to protect children from those who have repeatedly offended and are enforced in all 50 states and the District of Columbia (57, 58). Notification can include informing the public of a person’s status on the registry via flyers, phone calls, neighborhood meetings, or an Internet database. Similar to the sex offense registry, there is little research on the effectiveness of community notification (59). Although the public may report feeling safer due to these laws (60, 61), there has been no indication of increases in public safety because of these laws.

Ultimately, SORNA was created with the premise of keeping the public safe from those who commit repeat, violent sexual offenses and reduce sexual recidivism. However, in evaluating the effectiveness of SORN laws using current behavioral science literature, evidence does not support the use of SORN with AISB. In Levenson’s recent article outlining evidence-based recommendations for SORNA reform, she states, “Juveniles should not be labeled and defined for life by the single worst decision they might have made as a teenager” [(23), p. 4]. Extending SORNA to AISB is punitive and conflicts with the *parens patriae* principles of the juvenile justice system (62); therefore, these policies do not fall within a TJ philosophy. Within the TJ framework, policies should be informed by behavioral science research.

## Collateral consequences

SORN laws produced several unanticipated and unintended consequences, and researchers have begun to identify the unintended effects, known as *collateral consequences*, of SORN for AISB. The enforcement of SORN has severe, deleterious effects on AISB, their families, and the public. Hamilton (63) discussed the wide range of formal and informal collateral consequences of sex offense policies for individuals who have sexually offended and their families. In fact, treatment providers (62) and law enforcement (64) acknowledge the collateral consequences of SORN. Many professionals do not support SORN not only because of the lack of efficacy described earlier, but also due to the collateral consequences (65–68). Further, these laws have anti-therapeutic consequences on AISB, such as poor mental health outcomes, problems in community re-integration, and legal consequences, as described below.

### Mental health outcomes

In a survey of 265 treatment providers examining collateral consequences for AISB, providers noted juveniles subjected to SORN laws were much more likely to experience negative mental health outcomes and experience harassment than youth who were not subjected to registration requirements (62). Letourneau et al. (69) conducted a survey of 251 AISB across 18 states who were all receiving therapeutic services in outpatient settings for inappropriate, harmful, and/or illegal sexual behavior. Results indicated AISB who had to register were significantly more at-risk for negative outcomes, such as increased mental health symptoms, suicide attempts, problems in peer relations, and sexual abuse than non-registered AISB (69). Human Rights Watch (70) conducted extensive interviews of 296 individuals, comprised of individuals who committed offenses as children and their immediate family members, across 20 states. The report further documented the negative mental health consequences of being placed on the registry, including substance use and attempted or completed suicide.

### Familial problems

Research on AISB on the registry have noted the collateral consequences of SORNA extend beyond the AISB to their family members, causing financial social, and psychological distress (23, 47, 48, 70). There are several emotional, societal, and personal implications this process can have on the families of AISB. In the Human Rights Watch (70) report, 77% of the cases examined reported youth’s registration status had serious repercussions for their families and familial relationships, including increased financial burden, difficulties maintaining residence, and familial separation. Some family members of registered AISB lost their jobs because of the registration status of their family member. Fifty-two percent of the individuals reported experiencing violence or threats of violence against themselves or their family members which were directly related to being placed on the registry (70). Due to residency and travel restrictions enforced by SORNA, many families cannot relocate; limiting their ability to navigate financial difficulties.

### Community re-integration difficulties

AISB who are on the registry also have trouble maintaining stable housing when compared to youth who were not subjected to registration requirements (62). Residency restrictions, which exclude individuals adjudicated or convicted of a sex offense from living in certain distances from daycare centers, schools, or other areas where children congregate, are especially problematic for AISB. These restrictions have been enacted in at least 30 states and thousands of local municipalities (71) and often prohibit many from living within 1,000 or 2,500 feet from a school; in some urban areas, this can be quite difficult. These residency restrictions further impact individuals’ psychological well-being. In a study of adults who were subject to community notification, those who perceived more negative consequences due to notification and residence restrictions reported more elevated levels of depression and hopelessness (72). However, there is little evidence to support the notion that proximity to schools is related to sexual recidivism [see (73) for review]. Social science evidence has revealed these restrictions were enacted based on the fear that individuals who have been convicted of a sex offense would re-offend and do not make communities safer; therefore, TJ scholars have proposed residency restrictions be eliminated due to their anti-therapeutic effects (28).

AISB on the registry have more difficulties in school when compared to adolescents who were not subjected to registration requirements including barriers in returning to their schools (62). In fact, 52% of registered respondents in the Human Rights Watch survey noted they were denied access to, or experienced severe interruptions in, their primary or secondary education because of their registration status (70). For AISB who want to pursue college, there are further collateral consequences. Tewksbury and Lees (74) surveyed 26 adults listed on a sex offense registry maintained by a 4-year public college or university in the United States. Interestingly, though all the participants knew they were listed on the state registry, more than one-third were unaware they were listed on a university registry. More than half reported they were recognized at least a few times a year or more as a registrant, and others reported they were recognized daily. Participants also reported employment difficulties, including two-thirds reporting having lost or not received a job due to their registration status. Others reported housing difficulties, verbal and written harassment off-campus, and loss of friends.

By placing AISB on the registry, AISB experience the negative social effects of registering as a “sex offender” beyond the occupational and legal consequences. These labels lead to stigmatization, marginalization, fear of mistrust by others, harassment, and rejection or isolation by family and peers (11, 62, 70, 75). In fact, recommendations have been made to eliminate the use of the “juvenile sex offender” and “sex offender” labels (76–79). Essentially, these labels create a lifelong stigma which makes it difficult for AISB to successfully reintegrate back into their community.

### Legal consequences

SORN laws have enormous, long-lasting legal consequences on AISB aside from being placed on the public registry. Fees associated with registration can burden AISB and their families, with fees and costs associated with registration totaling between $800 and $2,000 per year (70). Failure-to-register convictions are the most common offense leading to reincarceration and sentencing for a single failure-to-register offense can be as long as 10 years in prison (70). Studies on failure-to-register noted the difficulty of maintaining registration and complying with the stringent guidelines for AISB (70). For instance, youth have trouble paying fees associated with registration, obtaining a proper residence or state identification card, and/or remembering when and where they need to register. Moreover, failure-to-register is not a significant predictor of sexual recidivism (70, 80), debunking the myth that individuals are more dangerous if they are noncompliant.

## Policy reform

Historically, legislation and policy has paid little attention to the developmental, social, and psychological factors that contribute to adolescents engaging in criminal behavior, including illegal sexual behavior. Many of the decisions the United States Supreme Court has made in the last 20 years concerning adolescents were based on brain development research and the Court acknowledged adolescents and adults are different in legally relevant ways (81, 82). The Court has issued rulings limiting the use of life without the possibility of parole in cases involving juveniles who have been convicted of serious crimes (*Graham v. Florida*, 2010), banning the use of capital punishment (*Roper v. Simmons*, 2005), and eliminating mandatory sentences of life without the possibility of parole (*Miller v. Alabama*, 2012; *Montgomery v. Louisiana*, 2016). Moreover, minoritized youth, such as LGBTQ youth, are disproportionately represented in the juvenile legal system and SORN laws have adverse consequences on these youth in community (83).

SORN policies for AISB do not examine adolescent behavior from a developmental life course perspective. Developmental psychology research has found adolescent cognition is present-oriented and lacks full decision-making and reasoning abilities (70, 82, 84). Adolescents engage in more impulsive, dangerous, risk-taking behaviors than adults, including risky sexual behaviors, because of their poor decision-making abilities (85). Therefore, many adolescents cannot fully understand the harmful consequences or wrongfulness of their behaviors (84). Adolescents’ rehabilitation can hinge on their ability to mature and age during the mandated treatment most receive. As a TJ framework encourages courts to incorporate behavioral science research, this literature on adolescent brain development should inform the courts about the implications related to SORNA. The downward extension to SORNA to AISB reflects the *adultification* of juveniles—a concept frequently used to describe how the system imparts adult responsibilities, behaviors, and treatment upon children and adolescents (86). Adultification ignores the developmental differences between adults and youth, often presuming inability to successfully rehabilitate. In order to adopt true TJ perspective, prosecutors, judges, and other stakeholders, must acknowledge these developmental differences in their application of the law.

**Figure 1:**
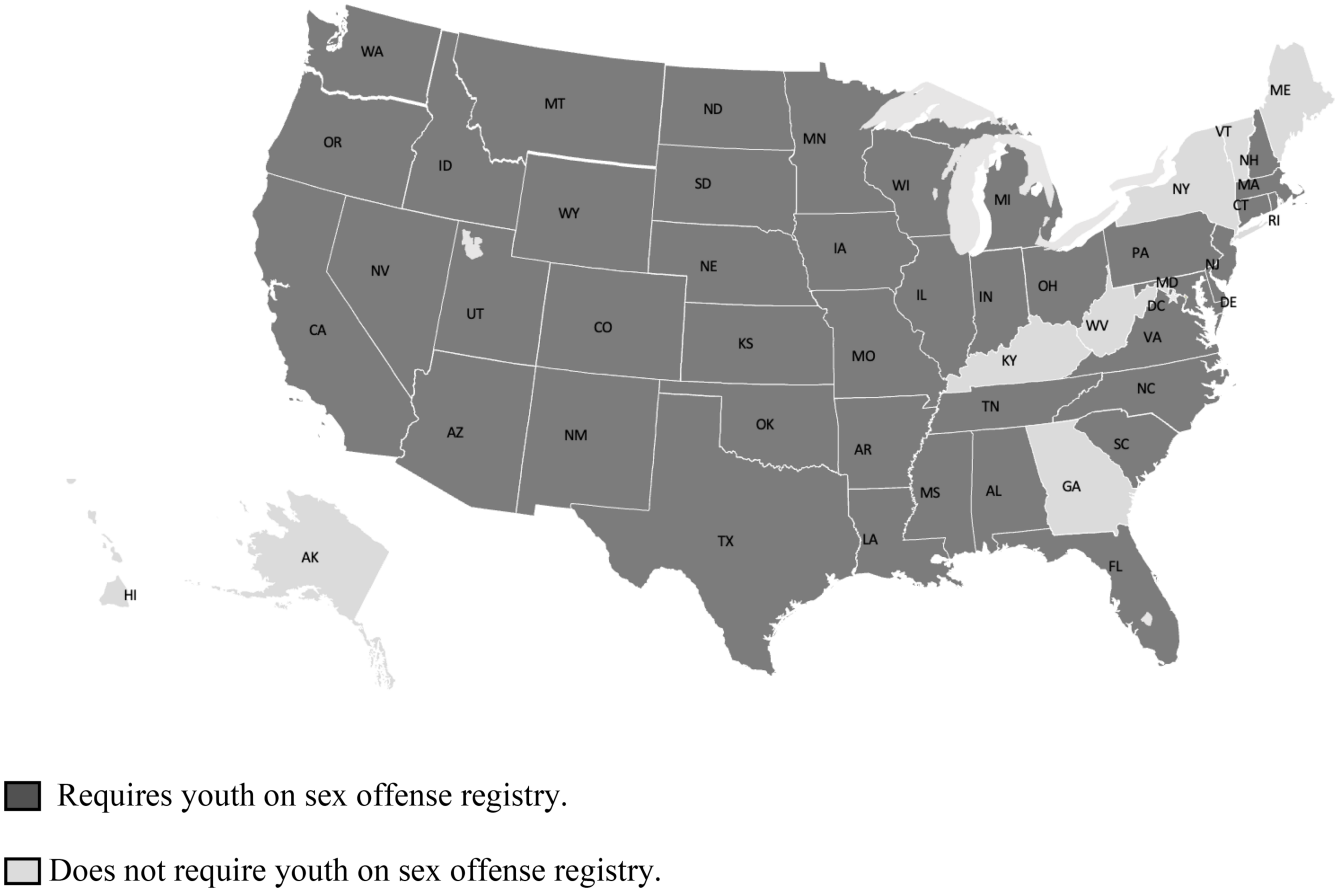
States requiring youth to be placed on sex offense registries (*N* = 42).

There are 42 states that require youth to be placed on the registry [see Figure 1; (87)]. Increasingly, states are beginning to remove lifetime registration requirements for AISB. On December 29, 2014, the Pennsylvania Supreme Court (in a 5–1 decision) issued a landmark ruling declaring that the lifetime registration requirements of SORN were unconstitutional as applied to AISB with certain sexual offenses (88). The decision held registration requirements violate AISB due process rights by utilizing the faulty presumption that pose a high risk of recidivism. On April 24, 2018, the New Jersey Supreme Court ended lifetime registration for AISB, citing rehabilitation and reformation remain hallmarks of the juvenile justice system (89). AISB will be able to petition the court to be removed from the registry after 15 years if they have not re-offended and are no longer a danger to the public. According to a TJ framework, lawmakers and those who apply the law (i.e., judge, probation officers) must be aware of the law’s effects on the mental health of all parties involved, including victims (90). Judicial discretion in these opinions appeared to adopt a TJ framework.

## Future directions and recommendations

Therapeutic jurisprudence should apply to AISB given the spirit of *parens patriae* in the juvenile courts and the reported rates of recidivism. The treatment of AISB in the juvenile justice system must be reformed to align with a TJ framework. Further, critics have argued that applying adult SORNA to juveniles “fails to consider the developmental and psychosocial contexts in which youth sexual offending occurs” [(62), p. 771]. Many researchers, mental health professionals, and advocates have stated juveniles should not be subjected to SORNA.

**Figure 2:**
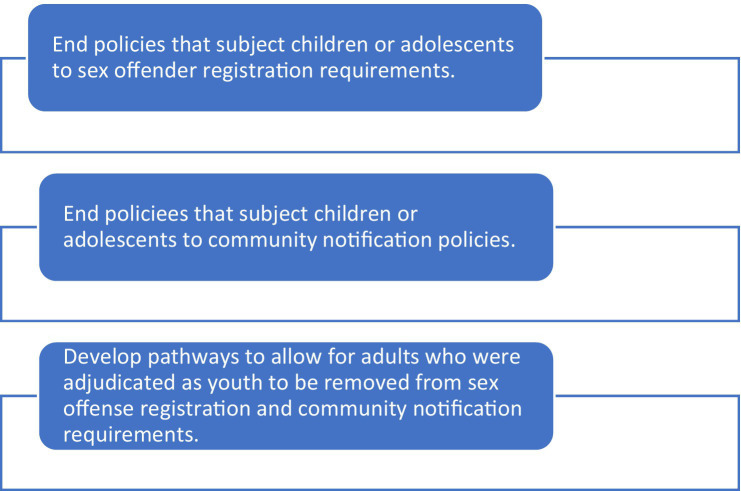
Policy recommendations.

Policies and legislation removing AISB from SORN requirements is ideal (see Figure 2). It is recommended that states end the use of the registry and community notification requirements for youth and adolescents. Further, mechanisms should be in place to allow for youth who are on the registry to be removed. Some states have legislation which allows for adolescents to be removed from the registry and allowing for removal is already built into the SORN requirements. For example, AISB who have lifetime registration currently can have their registration requirements terminated after 25 years if they have a “clean record.” At present, 29 states allow at least some individuals to petitions for removal (87). Implementing legislation which allows for clearer deregistration procedures for AISB currently on the registry, as well as adults who engaged in illegal sexual behavior as juveniles, would also align with the TJ framework. As noted previously, other countries, such as Canada (91), have non-public sex offense registries that only law enforcement can access. However, it should be noted that those registries also have little to no impact on community reintegration as well (92).

Instead of implementing SORNA, Edwards and Hensley (41) proposed a model of TJ which encourages *therapeutic management*, which supports community reintegration while maintaining and emphasizing community protection. From this approach, the therapeutic potential and needs of the adolescent can be identified early in the legal process, the youth and their family can be supported, and adequate post-treatment or post-release aftercare and supervision can be provided to allow for successful transition and reintegration. In Klotz’s conceptualization of TJ with adults who sexually offend, he suggests the therapeutic outcomes of the law on behavior, cognitive processes, and responsivity to treatment should be considered (42).

The present analysis informs two important recommendations for public policy reform regarding the application of SORN for AISB. First, youth should be exempt from public sex offense registration, community notification laws, and residency restriction laws using judicial discretion permitted by the Adam Walsh Act. Second, youth who are currently registered (or adults who were initially registered as youth) should be removed and no longer subject to community notification and residency restriction laws. States and local municipalities should ensure a clear and transparent deregistration process for these youth. The Human Rights Watch (70) explicitly recommends the United States should:

Amend state and federal law to explicitly exempt all persons who were below the age of 18 at the time of their offense from all sex offender registration, community notification, and residency restriction laws unless and until evidence-based research demonstrates that such requirements provide a significant, measurable improvement in public safety that outweighs the harms to former youth sex offenders and their families (p. 104).

## Conclusion

The aim of this paper was to analyze and critique the downward extension of SORN policies to AISB through a TJ framework. Therapeutic jurisprudence purports professionals should be mindful of the therapeutic and anti-therapeutic aspects of legal procedures, decisions, and policies. Further, this framework proposes evaluating how the law affects the psychological well-being of individuals who come into contact with the legal system. Therapeutic jurisprudence can assist professionals to balance the rights of those in the legal system and community safety (93). Lastly, TJ guides individuals to refute stereotypes and myths regarding individuals who have committed sex offenses with empirical data and consider the values of the principles of TJ (28).

Current policies on sexual offending in the United States are not only ineffective, but harmful for adolescents, which does not align with a TJ framework. Internationally, the International Association for the Treatment of Sexual Offenders (IATSO) has also noted that registries and community notification should not be applied to youth (94). The downward extension of SORNA to AISB is anti-therapeutic given the above collateral consequences on the youth and their families. The application of SORN for youth was driven by public misperceptions regarding AISB. SORN laws are harmful and anti-therapeutic to AISB given the collateral consequences, especially for AISB who successfully completed mandated treatment. When examining the application of SORN to AISB from a TJ framework, the primary task of TJ is “to identify—and ultimately to examine empirically—relationships between legal arrangements and therapeutic outcomes” [(30), p.32]. A TJ approach examines the way law and policies are implemented that can have an increase, decrease, or have a neutral effect on the well-being of those who have offended (38). There is well-documented research outlining the negative collateral consequences of SORN with AISB. Community notification laws have been categorized as “immoral, cruel and inhumane, and detrimental to the goal of reducing sexual offending” (28). The collateral consequences of SORN, community notification and residency restrictions can take place over an extensive period and be lifelong for many AISB and their families. The amount of time it requires for an adolescent and their family to rebuild and reconstruct their identity both within their respective structure and in the community is individualized and cannot be predicted. Given the rate of recidivism, while accounting for the development trajectory of adolescence, the use of SORN with adolescents is contraindicated. Further, SORN laws reinforce labeling and de-emphasizes the capacity for adolescents to rehabilitate (95).

Advocates in the juvenile justice system can help use the TJ framework to manage the dual role of protecting community rights and the rights of AISB by supporting the dignity and well-being of these adolescents (93). For TJ to be truly effective, the principles and supporting empirical data must be extended past academia and into real-world practice (34). Policymakers can use data to inform new statutes or modifications in existing policy (73). Campbell (96) discusses a need to shift from “evidence-based practice, to an evidence-based and evidence-informed approach to policymaking, to a TJ framework for evidence-informed health policymaking.” Through a TJ lens, Wexler further notes the task of TJ is “to determine how the law can use behavioral science information to improve therapeutic functioning without impinging upon concerns about justice” [(97), p. 280]. Reform efforts, such as implementing restorative justice, deregistering current adults who were adjudicated for illegal sexual behavior as minors, and eliminating SORN for AISB, are necessitated to fully encompass a TJ approach for AISB involved the juvenile justice system. Finally, other countries should refrain from incorporating public registries given the extensive literature documenting the ineffectiveness and collateral consequences of such public registries in the United States.

## Author contributions

AA and JF contributed to the conception of the project and wrote the draft of the manuscript. CM, EF, and MJ reviewed the manuscript and provided feedback on content. AA coordinated inputs from all authors. All authors contributed to the article and approved the submitted version.

## Conflict of interest

The authors declare that the research was conducted in the absence of any commercial or financial relationships that could be construed as a potential conflict of interest.

## Publisher’s note

All claims expressed in this article are solely those of the authors and do not necessarily represent those of their affiliated organizations, or those of the publisher, the editors and the reviewers. Any product that may be evaluated in this article, or claim that may be made by its manufacturer, is not guaranteed or endorsed by the publisher.

## References

[1] Finkelhor D. Ormrod R. Chaffin M. Juveniles who commit sex offenses against minors. (2009). Available at: http://unh.edu/ccrc/pdf/CV171.pdf

[2] Christiansen AK Vincent JP. Characterization and prediction of sexual and nonsexual recidivism among adjudicated juvenile sex offenders. Behav Sci Law. (2013) 31:506–29. doi: 10.1002/bsl.2070, PMID: 23703937

[3] Burton DL. Were adolescent sexual offenders children with sexual behavior problems? Sex Abuse. (2000) 12:37–48. doi: 10.1177/107906320001200105 10729958

[4] Borduin CM Schaeffer CM Heiblum N. A randomized clinical trial of multi-systemic therapy with juvenile sexual offenders: effects on youth social ecology and criminal activity. J Consult Clin Psychol. (2009) 77:26–37. doi: 10.1037/a0013035, PMID: 19170451

[5] Caldwell MF. Sexual offense adjudication and sexual recidivism among juvenile offenders. Sex Abuse. (2007) 19:107–13. doi: 10.1177/107906320701900203, PMID: 17530405

[6] Reitzel LR Carbonell JL. The effectiveness of sexual offender treatment for juveniles as measured by recidivism: a meta-analysis. Sex Abuse. (2006) 18:401–21. doi: 10.1007/s11194-006-9031-2, PMID: 17136623

[7] Schmidt SR Bonner BL Chaffin M. Understanding and treating adolescents with illegal sexual behavior In: Goodyear-Brown P, editor. Handbook of child sexual abuse: Identification, assessment, and treatment. Hoboken, NJ: John Wiley & Sons (2012)

[8] Caldwell MF. Quantifying the decline in juvenile sexual recidivism rates. Psychol Public Policy Law. (2016) 22:414–26. doi: 10.1037/law0000094

[9] Caldwell MF. What we do not know about juvenile sexual reoffense risk. Child Maltreat. (2002) 7:291–302. doi: 10.1177/107755902237260, PMID: 12408242

[10] Rasmussen LAL. Youth adjudicated for sex offenses, followed into adulthood, and found on a state sex offender registry. J Aggress Maltreat Trauma. (2022) 31:1359–78. doi: 10.1080/10926771.2022.2112332

[11] Chaffin M. Our minds are made up—Don’t confuse us with the facts: commentary on policies concerning children with sexual behavior problems and juvenile sex offenders. Child Maltreat. (2008) 13:110–21. doi: 10.1177/1077559508314510, PMID: 18408208

[12] Quinn JF Forsyth CJ Mullen-Quinn C. Societal reaction to sex offenders: a review of the origins and results of the myths surrounding their crimes and treatment amenability. Deviant Behav. (2004) 25:215–32. doi: 10.1080/01639620490431147

[13] Cain CM Sample LL. Public opinions on applying adult sex offender legislation to minors convicted of sex crimes. Crim Justice Policy Rev. (2022) 33:235–55. doi: 10.1177/08874034211046327

[14] Cain CM Sample LL Anderson AL. Public opinion of the application of sex offender notification laws to female sex offenders: why it is important to examine. Crim Justice Policy Rev. (2017) 28:155–75. doi: 10.1177/00887403415572253

[15] Connor DP Tewksbury R. Public and professional views of sex offender registration and notification. Criminology Criminal Justice Law Soc. (2017) 18:1–27.

[16] Kernsmith PD Craun SW Foster J. Public attitudes toward sexual offenders and sex offender registration. J Child Sex Abus. (2009) 18:290–301. doi: 10.1080/10538710902901663, PMID: 19856734

[17] Levenson JS Brannon YN Fortney T Baker J. Public perceptions about sex offenders and community protection policies. Anal Soc Issues Public Policy. (2007). 137–161. doi: 10.1111/j.1530-2415.2007.00119.x

[18] Gavin H. The social construction of the child sex offender explored by narrative. Qual Rep. (2005) 10:395–415.

[19] Hinds L Daly K. The war on sex offenders: community notification in perspective. Aust N Z J Criminol. (2000) 13:284–306.

[20] Sample LL. An examination of the degree to which sex offenders kill. Crim Justice Rev. (2006) 31:230–50. doi: 10.1177/0734016806292929

[21] Zgoba KM. Spin doctors and moral crusaders: the moral panic behind child safety legislation. Crim Justice Stud. (2004) 17:385–404. doi: 10.1080/1478601042000314892

[22] Fix RL Cyperski MA Burkhart BR. Disproportionate minority contact comparisons across juveniles adjudicated for sexual and non-sexual offenses. Sex Abuse. (2015) 29:291–308. doi: 10.1177/1079063215601436 26297505

[23] Levenson JS. Sex offender management policies and evidence-based recommendations for registry reform. Curr Psychiatry Rep. (2018) 20:21–7. doi: 10.1007/s11920-018-0884-0, PMID: 29560559

[24] Lussier P Mathesius J. Not in my backyard: public sex offender registries and public notification laws. Can J Criminol Crim Justice. (2019) 61:105–16. doi: 10.3138/cjccj.2018-0026

[25] Griffin G Jenuwine MJ. Using therapeutic jurisprudence to bridge the juvenile justice and mental health systems. Univ Cincinnati Law Rev. (2002) 71:65–87.

[26] Hernández MC. Mentally ill in the juvenile justice system: the sequential intercept model approach. Int J Ther Jurisprudence. (2017) 2:61–93.

[27] Wexler DB. Inducing therapeutic compliance through the criminal law. Law Psychol Rev. (1990) 14:43–57.

[28] Cucolo HE Perlin ML. Preventing sex-offender recidivism through therapeutic jurisprudence approaches and specialized community integration. Temple Political Civil Rights Law Rev. (2012) 1:1–42. doi: 10.2139/ssrn.2116424

[29] Perlin ML. Therapeutic jurisprudence and outpatient commitment law: Kendra’s law as a case study. Psychol Public Policy Law. (2003) 9:183–208. doi: 10.1037/1076-8971.9.1-2.183 16700141

[30] Wexler DB. Putting mental health into mental health law: therapeutic jurisprudence. Law Hum Behav. (1992) 16:27–38. doi: 10.1007/BF02351047

[31] Perlin ML. “Yonder stands your orphan with his gun”: the international human rights and therapeutic jurisprudence implications of juvenile punishment schemes. Texas Tech Law Rev. (2013) 46:301–38.

[32] Winick BJ. Outpatient commitment: a therapeutic jurisprudence analysis. Psychol Public Policy Law. (2003) 9:107–44. doi: 10.1037/1076-8971-9.1-2.107 16700139

[33] King MS. Restorative justice, therapeutic jurisprudence, and the rise of emotionally intelligent justice. Melbourne University Law Rev. (2008) 32:1096–126.

[34] Perlin ML. What is therapeutic jurisprudence? New York Law School J Human Rights. (1993) 10:623–36.

[35] Perlin ML. “Changing of the guards”: David Wexler, therapeutic jurisprudence, and the transformation of legal scholarship. Int J Law Psychiatry. (2019) 63:3–7. doi: 10.1016/j.ijlp.2018.07.001, PMID: 30031566

[36] SMART Office: Office of Sex Offender Sentencing, Monitoring, Apprehending, Registering, and Tracking. (2016). Available at: https://www.smart.gov/about.htm

[37] Office of Sex Offender Sentencing, Monitoring, Apprehending, Registering and Tracking. (2017). Available at: https://www.smart.gov/pdfs/SORNA-progress-check.pdf

[38] Birgden A. Therapeutic jurisprudence and sex offenders: a psycho-legal approach to protection. Sex Abuse. (2004) 16:351–64. doi: 10.1177/107906320401600407, PMID: 15560416

[39] Birgden A. Serious sex offenders monitoring act 2005 (Vic): a therapeutic jurisprudence analysis. Psychiatry Psychol Law. (2007) 14:78–94. doi: 10.1375/pplt.14.1.78

[40] Birgden A Cucolo H. The treatment of sex offenders: evidence, ethics, and human rights. Sex Abuse. (2011) 23:295–313. doi: 10.1177/1079063210381412 20937793

[41] Edwards W Hensley C. Restructuring sex offender sentencing: a therapeutic jurisprudence approach to the criminal justice process. Int J Offender Ther Comp Criminol. (2001) 45:646–62. doi: 10.1177/0306624X01456002

[42] Klotz JA. Sex offenders and the law: new directions In: Wexler DB Winick BJ, editors. Law in a therapeutic key: developments in therapeutic jurisprudence. Durham, NC: Carolina Academic Press (1996). 131–43.

[43] Winick BJ. Sex offender laws in the 1990’s: a therapeutic jurisprudence analysis. Public Policy Psychol Law. (1998) 4:505–70. doi: 10.1037/1076-8971.4.1-2.505

[44] Letourneau EJ Levenson JS Bandyopadhyay D Sinha D Armstrong KS. Effects of South Carolina’s sex offender registration and notification policy on adult recidivism. Crim Justice Policy Rev. (2010) 21:435–58. doi: 10.1177/0887403409353148

[45] Levenson JS Grady MD Leibowitz G. Grand challenges: social justice and the need for evidence-based sex offender registry reform. J Sociol Soc Welf. (2016) 43:3–38.

[46] Pittman N Nguyen Q. A snapshot of juvenile sex offender registration and notification laws: a survey of the United States. Philadelphia, PA: Defender Association of Philadelphia (2011) http://www.njjn.org/uploads/digital-library/SNAPSHOT_web10-28.pdf.

[47] Letourneau EJ Miner MH. Juvenile sex offenders: a case against the legal and clinical status quo. Sex Abus. (2005) 17:293–312. doi: 10.1177/107906320501700304, PMID: 16121840

[48] Zimring FE. An American travesty: legal responses to adolescent sexual offending. Chicago, IL: The University of Chicago Press (2004).

[49] Falligant JM Alexander AA Burkhart BR. Risk assessment of juveniles adjudicated for possession of child sexual exploitation material. J Forensic Psychol Res Pract. (2017) 17:145–56. doi: 10.1080/15227932.2017.1270640

[50] Caldwell MF Ziemke MH Vitacco MJ. An examination of the sex offender registration and notification act as applied to juveniles: evaluating the ability to predict sexual recidivism. Psychol Public Policy Law. (2008) 14:89–114. doi: 10.1037/a0013241

[51] Harris AJ Lobanov-Rostovsky C Levenson JS. Widening the net: the effects of transitioning to the Adam Walsh Act’s federally mandated sex offender classification system. Crim Justice Behav. (2010) 37:503–19. doi: 10.1177/0093854810363889

[52] Letourneau EJ Armstrong KS. Recidivism rates for registered and nonregistered juvenile sex offenders. Sex Abuse. (2008) 20:393–408. doi: 10.1177/1079063208324661, PMID: 18948430

[53] Letourneau EJ Bandyopadhyay D Sinha D Armstrong K. Effects of sex offender registration policies on juvenile justice decision making. Sex Abuse. (2009) 21:149–65. doi: 10.1177/1079063208328678, PMID: 19141629

[54] Zimring FE Piquero AR Jennings WG. Sexual delinquency in Racine: does early sex offending predict later sex offending in youth and young adulthood? Criminol Public Policy. (2007) 6:507–34. doi: 10.1111/j.1745-9133.2007.00451.x

[55] Batastini AB Hunt E Present-Koller J DeMatteo D. Federal standards for community registration of juvenile sex offenders: An evaluation of risk prediction and future implications. Psychology, Public Policy, and Law. (2011) 17:451–74. doi: 10.1037/a0023637, PMID: 19141629

[56] Caldwell MF Dickinson C. Sex offender registration and recidivism risk in juvenile sex offenders. Behavioral Sciences & the Law. (2009) 27:941–56. doi: 10.1002/bsl.907 19937920

[57] Cohen M Jeglic EL. Sex offender legislation in the United States. Int J Offender Ther Comp Criminol. (2007) 51:369–83. doi: 10.1177/0306624X06296235 17652143

[58] Levenson JS. Policy interventions designed to combat sexual violence: community notification and civil commitment. J Child Sex Abus. (2003) 12:17–52. doi: 10.1300/J070v12n03_02, PMID: 15308446

[59] Zevitz RG Farkas MA. Sex offender community notification: managing high risk criminals or exacting further vengeance? Behav Sci Law. (2000) 18:375–91. doi: 10.1002/1099-0798(200003/06)18:2/3<375::AID-BSL380>3.0.CO;2-N 10874294

[60] Anderson AL Sample LL. Public awareness and action resulting from sex offender community notification laws. Crim Justice Policy Rev. (2008) 19:371–96. doi: 10.1177/0887403408316705

[61] Beck VS Travis LF. Sex offender notification and protective behavior. Violence Vict. (2004) 19:289–302. doi: 10.1891/vivi.19.3.289.65762, PMID: 15631282

[62] Harris AJ Walfield SM Shields RT Letourneau EJ. Collateral consequences of juvenile sex offender registration and notification: results from a survey of treatment providers. Sex Abuse. (2016) 28:770–90. doi: 10.1177/1079063215574004, PMID: 25733541

[63] Hamilton E. Toward a focused conceptualization of collateral consequences among individuals who sexually offend: a systematic review. Sex Abus. (2022) 34:3–23. doi: 10.1177/1079063220981906, PMID: 33356891

[64] Cubellis MA Walfield SM Harris AJ. Collateral consequences and effectiveness of sex offender registration and notification: law enforcement perspectives. Int J Offender Ther Comp Criminol. (2018) 62:1080–106. doi: 10.1177/0306624X16667574, PMID: 27634816

[65] Mustaine EE. Sex offender residency restrictions: successful integration or exclusion? Criminol Public Policy. (2014) 13:169–77. doi: 10.1111/1745-9133.12076

[66] Payne BK Tewksbury R Mustaine EE. Identifying the sources of community corrections professionals’ attitudes about sex offender residence restrictions: the impact of demographics and perceptions. Crime Delinq. (2016) 62:143–68. doi: 10.1177/0011128712470993

[67] Tewksbury R Mustaine EE. Stress and collateral consequences for registered sex offenders. J Public Manag Soc Policy. (2009) 15:215–39.

[68] Tewksbury R Mustaine EE. Parole board members’ views of sex offender registration and community notification. Am J Crim Justice. (2012) 37:413–31. doi: 10.1007/s12013-011-9119-1

[69] Letourneau EJ Harris AJ Shields RT Walfield SM Ruzicka AE Buckman C. Effects of juvenile sex offender registration on adolescent well-being: an empirical examination. Psychol Public Policy Law. (2018) 24:105–17. doi: 10.1037/law0000155

[70] Human Rights Watch. Raised on the registry: the irreparable harm of placing children on sex offender registries in the U.S. Washington, DC: Human Rights Watch (2013).

[71] Meloy ML Miller SL Curtis KM. Making sense out of nonsense: the deconstruction of state-level sex offender residence restrictions. Am J Crim Justice. (2008) 33:209–22. doi: 10.1007/s12103-008-9042-2

[72] Jeglic EL Mercado CC Levenson JS. The prevalence and correlates of depression and hopelessness among sex offenders subject to community notification and residence restriction legislation. Am J Crim Justice. (2012) 37:46–59. doi: 10.1007/s12103-010-9096-9

[73] Chajewski M Mercado CC. An evaluation of sex offender residency restriction functioning in town, county, and city-wide jurisdictions. Crim Justice Policy Rev. (2009) 20:44–61. doi: 10.1177/0887403408320845

[74] Tewksbury R Lees M. Perceptions of sex offender registration: Collateral consequences and community experiences. Sociological Spectrum. (2006) 26:309–34. doi: 10.1080/02732170500524246

[75] Bailey DJS Klein JL. Ashamed and alone: comparing offender and family member experiences with the sex offender registry. Crim Justice Rev. (2018) 43:440–57. doi: 10.1177/0734016818756486

[76] Fanniff AM Alexander AA. Improving justice, equity, diversity, and inclusion in research on sexual abuse perpetration. Sex Abus. (2022) 34:780–805. doi: 10.1177/10790632221091193, PMID: 35548859

[77] Harris AJ Socia KM. What’s in a name? Evaluating the effects of the “sex offender” label on public opinions and beliefs. Sex Abuse. (2016) 28:660–78. doi: 10.1177/1079063214564391, PMID: 25542837

[78] Willis GM. Why call someone by what we don’t want them to be? The ethics of labeling in forensic/correctional psychology. Psychol Crime Law. (2018) 24:727–43. doi: 10.1080/1068316X.2017.1421640

[79] Willis GM Letourneau EJ. Promoting accurate and respectful language to describe individuals and groups. Sex Abus. (2018) 30:480–3. doi: 10.1177/1079063218783799, PMID: 29998798

[80] Zgoba K Levenson J. Failure to register as a predictor of sex offense recidivism: the big bad wolf or a red herring. Sex Abuse. (2012) 24:328–49. doi: 10.1177/1079063211421019, PMID: 22138613

[81] Steinberg L. Should the science of adolescent brain development inform public policy? Am Psychol. (2009) 64:739–50. doi: 10.1037/0003-066X.64.8.739 19899880

[82] Steinberg L. Adolescent brain science and juvenile justice policymaking. Psychol Public Policy Law. (2017) 23:410–20. doi: 10.1037/law0000128

[83] Janseen J DeMatteo D. The application of mercy: equal treatment for all youth who commit sex offenses. Mitchell Hamline Law Rev. (2020) 46:344–66.

[84] Steinberg L Scott ES. Less guilty by reason of adolescence: developmental immaturity, diminished responsibility, and the juvenile death penalty. Am Psychol. (2003) 58:1009–18. doi: 10.1037/0003-066X.58.12.1009, PMID: 14664689

[85] Steinberg L. A social neuroscience perspective on adolescent risk-taking. Dev Rev. (2008) 28:78–106. doi: 10.1016/j.dr.2007.08.002, PMID: 18509515 PMC2396566

[86] Arnett C. Virtual shackles: electronic surveillance and the adultification of juvenile courts. J Criminal Law Criminology. (2018) 108:399–454.

[87] Juvenile Law Center. Labeled for life: a review of youth sex offender registration laws. (2020). Available at: https://jlc.org/sites/default/files/attachments/2020-08/JLC_SORNA-Report_8-15_0.pdf

[88] In re J.B. 107 A.3d 1 Pa (2014).

[89] State of New Jersey in the Interest of C.K. (A-15-16) (077672) NJ (2018).

[90] Wemmers J. Victim participation and therapeutic jurisprudence. Vict Offenders. (2008) 3:165–91. doi: 10.1080/15564880801938318

[91] Murphy L Fedoroff JP Martineau M. Canada’s sex offender registries: background, implementation, and social policy considerations. Can J Hum Sex. (2009) 18:61–72.

[92] Murphy L Fedoroff JP. Sexual offenders’ views of Canadian sex offender registries: a survey of a clinical sample. Can J Behav Sci. (2013) 45:238–49. doi: 10.1037/a0033251

[93] Birgden A Perlin ML. “Where the home in the valley meets the damp dirty prison”: A human rights perspective on therapeutic jurisprudence and the role of forensic psychologists in correctional settings. Aggression and Violent Behavior. (2009) 14:256–63. doi: 10.1016/j.avb.2009.04.002

[94] Miner M Borduin C Prescott D Bovensmann H Schepker R Du Bois R. Standards of care for juvenile sexual offenders of the International Association for the Treatment of sexual offenders. Sex Offender Treat. (2006) 1:1–7.

[95] Birgden A Vincent F. Maximizing therapeutic effects in treating sexual offenders in an Australian correctional system. Behav Sci Law. (2000) 18:479–88. doi: 10.1002/1099-0798(2000)18:4<479::AID-BSL388>3.0.CO;2-J, PMID: 11018780

[96] Campbell AT. Therapeutic jurisprudence: a framework for evidence-informed health care policymaking. Int J Law Psychiatry. (2010) 33:281–92. doi: 10.1016/j.ijlp.2010.09.001, PMID: 20888646

[97] Wexler DB. Therapeutic jurisprudence and the criminal courts. William Mary Law Rev. (1993) 35:279–99.

